# Supported Intermetallic PdZn Nanoparticles as Bifunctional Catalysts for the Direct Synthesis of Dimethyl Ether from CO‐Rich Synthesis Gas

**DOI:** 10.1002/anie.201906256

**Published:** 2019-09-19

**Authors:** Manuel Gentzen, Dmitry E. Doronkin, Thomas L. Sheppard, Anna Zimina, Haisheng Li, Jelena Jelic, Felix Studt, Jan‐Dierk Grunwaldt, Jörg Sauer, Silke Behrens

**Affiliations:** ^1^ Institute of Catalysis Research and Technology Karlsruhe Institute of Technology (KIT) Herrmann-von-Helmholtz-Platz 1 76344 Eggenstein-Leopoldshafen Germany; ^2^ Institute for Chemical Technology and Polymer Chemistry Karlsruhe Institute of Technology (KIT) Engesserstr. 20 76131 Karlsruhe Germany; ^3^ School of Physics and Engineering Henan University of Science and Technology 471023 Luoyang, Henan Province P. R. China

**Keywords:** bifunctional catalysts, density functional calculations, dimethyl ether synthesis, operando XAS, Pd/Zn nanoparticles

## Abstract

The single‐step syngas‐to‐dimethyl ether (STD) process entails economic and technical advantages over the current industrial two‐step process. Pd/ZnO‐based catalysts have recently emerged as interesting alternatives to currently used Cu/ZnO/Al_2_O_3_ catalysts, but the nature of the active site(s), the reaction mechanism, and the role of Pd and ZnO in the solid catalyst are not well established. Now, Zn‐stabilized Pd colloids with a size of 2 nm served as the key building blocks for the methanol active component in bifunctional Pd/ZnO‐γ‐Al_2_O_3_ catalysts. The catalysts were characterized by combining high‐pressure operando X‐ray absorption spectroscopy and DFT calculations. The enhanced stability, longevity, and high dimethyl ether selectivity observed makes Pd/ZnO‐γ‐Al_2_O_3_ an effective alternative system for the STD process compared to Cu/ZnO/γ‐Al_2_O_3_.

## Graphical abstract

**Zinc‐stabilized palladium colloids** with a size of about 2 nm are the key building units for bifunctional syngas‐to‐dimethyl ether catalysts with enhanced stability, longevity, and high dimethyl ether selectivity. The catalysts were characterized by combining high‐pressure operando X‐ray absorption spectroscopy and DFT calculations.

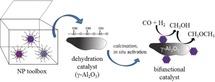

Dimethyl ether (DME) is industrially produced by dehydration of methanol derived from synthesis gas (CO/H_2_), with a global production of 5 Mtons/year.^1^ This two‐step process typically employs Cu/ZnO/Al_2_O_3_ catalysts for methanol synthesis and solid acids (for example, γ‐Al_2_O_3_ or zeolites) for methanol dehydration. The development of an alternative single‐step STD process is highly desirable as it entails economic and technical advantages, for example, cost saving by process simplification and higher syngas conversions.^1^ This necessitates the design of suitable bifunctional catalysts with high methanol synthesis and dehydration activity. Recent efforts have involved preparing physical mixtures of the two components^2^ as well as bifunctional catalysts,^3^ which couple the two sequential reactions on active sites in close proximity to each other. Conventional Cu/ZnO‐based methanol catalysts have been one focus of study; however, these systems can be prone to deactivation, which drives the search for new catalysts.^4^ Recently, Pd‐based intermetallic compounds have emerged as interesting alternatives providing improved methanol selectivity and thermal stability. Pd/ZnO systems in particular have been successfully employed in methanol steam reforming (MSR)^5^ and the water‐gas‐shift (WGS) reaction,^6^ and have recently proven to be a suitable methanol synthesis component for the STD process in combination with a solid acid.3b, 7 The activity of these catalysts was ascribed to the formation of intermetallic PdZn during the reductive catalyst pretreatment.3b, 6, 8

While Pd/ZnO‐based catalysts have been investigated experimentally and theoretically, for example, for MSR and WGS, the exact nature of the active site(s) and the reaction mechanism for bifunctional Pd/ZnO based STD catalysts is not well defined, and depends strongly on the synthesis route employed.3b, 7, 9 Herein we focus on the colloidal synthesis of highly uniform Pd/Zn‐based nanoparticles (NPs). This method allows for evenly distributed metal content, sizes, and shapes, which are ideal for mechanistic investigation using complementary spectroscopy, and computational modeling.^10^ We show how Pd/Zn catalyzes the hydrogenation of CO to DME by employing high‐pressure operando X‐ray absorption spectroscopy (XAS), in conjunction with density functional theory (DFT) calculations.

Uniform Pd/Zn‐based NPs were synthesized by reacting Pd(acac)_2_ and Et_2_Zn, yielding particles with the Pd core in close contact to the Zn‐containing organic shell without the need for additional ligands or surfactants (Supporting Information). Since Pd^0^ promotes the formation of methane over methanol, it is critical to avoid the formation of Pd^0^ via reactive Pd^0^‐Zn^2+^ interfaces by ensuring the sufficient supply of Zn atoms during catalyst preparation and activation. This synthesis concept is versatile and provides various NP building blocks for the methanol‐active component.10a–10c TEM images revealed highly uniform Pd/Zn NPs of 2.0±0.4 nm diameter (Figure 1 a). The molar Pd:Zn ratio of the NP powder was 1:2.9, in agreement with the Pd(acac)_2_:Et_2_Zn ratio used during synthesis. After deposition on γ‐Al_2_O_3_ and calcination, two bifunctional STD catalysts with Pd loadings of 8.4 wt % (Pd/Zn (8)‐γ‐Al_2_O_3_) and 14.3 wt % (Pd/Zn (14)‐γ‐Al_2_O_3_) were obtained (see the Supporting Information, Table S1, for elemental composition, specific surface area, number of acid sites).

**Figure 1:**
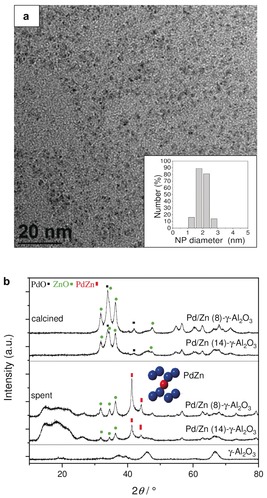
a) TEM image of the Pd/Zn NPs (inset: particle size distribution). b) XRD patterns of the calcined and spent Pd/Zn‐based STD catalysts (inset: PdZn structure (*P*4/*mmm*; Pd blue, Zn red).

X‐ray diffraction (XRD) of the calcined STD catalysts showed reflections of PdO, Pd, ZnO, and γ‐Al_2_O_3_ (Figure 1 b) and no reflections from mixed oxides or alloyed species. Following catalytic tests, the spent catalysts revealed reflections at 41.2° and 44.1° (2*θ*) characteristic of the L1_0_ PdZn phase, respectively, and no Pd^0^ reflections (Figure 1 b). Topotactic alloy formation involves considerable reduction of ZnO in close contact to a noble metal capable of dissociative H_2_ adsorption (that is, Pd). Since NP synthesis afforded a molar Pd/Zn ratio of 1:3, sufficient Zn was available for complete formation of an intermetallic PdZn phase. Residual Zn^2+^ species remained as ZnO in the spent STD catalyst. According to the Scherrer equation, particle sizes were 8–11 nm for PdO and 16 nm for PdZn in the calcined and spent catalysts, respectively. TEM and SEM images of the calcined and spent catalysts revealed Pd/Zn‐based NPs well distributed over the Al_2_O_3_ dehydration catalyst (Supporting Information, Figure S1), facilitating close proximity between the consecutive catalytic functions of methanol synthesis and dehydration.

The catalytic properties of the bifunctional PdZn catalysts in the STD reaction were determined in a fixed‐bed reactor (50 bar, 250–300 °C, 70 vol % inert gas (Ar/N_2_); Supporting Information, Table S2) using syngas with a H_2_/CO composition of 1:1, simulating that derived from renewable biomass feedstock. For Pd/Zn (8)‐γ‐Al_2_O_3_, CO conversion increased with temperature until a maximum at 290 °C (*X*_CO_ 37 %; Figure 2 b). At higher temperatures, thermodynamic equilibrium constraints become more important, resulting in decreased CO conversion of 34 % at 300 °C.

**Figure 2:**
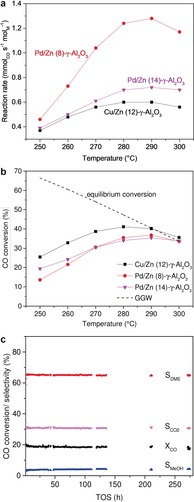
a) Reaction rates and b) CO conversions for STD catalysts derived from Pd/Zn and Cu/Zn NPs (50 bar, H_2_:CO=1, 70 vol % inert gases). c) Conversion and selectivity with TOS (250 °C) (Pd/Zn (8)‐γ‐Al_2_O_3_).

PdZn alloys showed good selectivity to DME while maintaining high activity (*S*_DME_ 65 % at 250–270 °C, *S*_DME_ 62 % at 300 °C). CH_4_ formation was negligible between 250 °C and 280 °C (*S*_CH4_<1 %) and increased only slightly to 2.4 % at 300 °C. In contrast, Pd_2_Ga NPs showed a lower DME and a higher CH_4_ selectivity.10c The ratio of methanol to dehydration catalyst is also known to influence catalytic performance.^11^ Interestingly, the reaction rate normalized to amount of Pd (in mol) was superior for Pd/Zn (8)‐γ‐Al_2_O_3_ compared to Pd/Zn (14)‐γ‐Al_2_O_3_ illustrating the influence of well‐balanced catalyst functionalities. Pd/Zn (14)‐γ‐Al_2_O_3_ revealed S_MeOH_ of 9 % (250 °C) indicating incomplete methanol dehydration, which is probably due to the lower γ‐Al_2_O_3_ content (*S*_MeOH_ 3 % for Pd/Zn (8)‐γ‐Al_2_O_3_; Supporting Information, Tables S1, S2; Figure S2). Therefore, less methanol was withdrawn from the equilibrium, and lower reaction rates compared to Pd/Zn (8)‐γ‐Al_2_O_3_ were observed. Compared to the Cu/Zn‐based reference catalyst prepared via a similar synthetic approach, Pd/Zn catalysts showed enhanced CO turnover rates and less CH_4_ formation (Figure 2 a; Supporting Information, Figure S2).10a Most notably, the Pd‐based catalysts showed excellent stability over time on stream (TOS; Figure 2 c), with CO conversion and DME selectivity remaining constant after 270 h. After testing the catalyst at temperatures up to 300 °C, *X*_CO_ was reproducible at 250 °C. In contrast, the Cu/Zn‐based reference catalyst revealed progressive deactivation (Supporting Information, Figure S3).10a Compared to Cu, both higher thermal stability of the intermetallic PdZn compound and higher hydrogenation activity of Pd may prevent particles sintering and deposition of carbonaceous species under these conditions, respectively.

Formation of the active catalyst phase under reducing H_2_ atmosphere was monitored by in situ XAS at pressures up to 20 bar. XAS spectra (Figure 3 a,b) recorded at room temperature approximately 3 min after switching to reducing H_2_ flow showed complete reduction of PdO in the calcined catalyst (Figure 3 c), while ZnO remained oxidized (Figure 3 b,d; reference spectra, Supporting Information, Figure S3). The reduction of PdO_*x*_ NPs with H_2_ at low temperatures has been previously reported for Pd‐based catalysts.^12^ The X‐ray absorption near edge structure (XANES) spectrum (20 °C, 5 % H_2_/He) showed a circa 0.8 eV shift of the rising edge to higher energies and shifts of all maxima to lower energies compared to the Pd^0^ reference spectrum (Figure 3 a). Since no reduction of Zn occurred at 20 °C, the observed shifts of the XANES features of Pd NPs could be attributed only to formation of PdH_*x*_ hydrides.^13^ While extended X‐ray absorption fine structure (EXAFS) is not sensitive to light atoms like hydrogen, Pd K edge XANES may be used to detect changes in the structure of unoccupied electronic states and is thus affected by the formation of PdH_*x*_ owing to mixing of unoccupied states of hydrogen and palladium.14a The observed shifts of the XANES features and the difference spectrum compared to metallic Pd (Supporting Information, Figure S4c) are consistent with the formation of PdH_*x*_ NPs.^14^ They disappeared at 45 °C confirming decomposition of PdH_*x*_ to Pd^0^ species (inset in Figure 3 a; Supporting Information, Figure S4d). The Pd K edge position continuously shifted to lower energies during heating, probably caused by alloying of Pd with Zn via spillover of hydrogen from PdH_*x*_ NPs to the neighboring ZnO species.^13^ No significant changes in the Pd K spectra occurred above 180 °C (Supporting Information, Figure S5a). Linear combination analysis (LCA, Supporting Information, Figure S5b) of the Zn K edge XANES spectra showed that a Pd^0^:Zn^0^ ratio of 1:1 was reached at 180 °C. The EXAFS region in the Pd K edge spectra exhibited pronounced changes until 250 °C, indicating alloying with Zn (Figure 3 a). Gradual reduction of Zn was also observed during activation up to 250 °C (Figure 3 b), where 31 % (±10 %)^15^ of ZnO was reduced to Zn^0^. Following activation, the atomic Zn^0^ to Pd^0^ ratio was 1.3:1, suggesting the formation of alloyed PdZn NPs with an excess of Zn^0^ species, probably on the surface of PdZn, favored by lower surface energy of Zn. This is further supported by the Fourier‐transformed (FT) EXAFS (Figure 3 c,d). At the Pd K edge, EXAFS revealed bond distances of 2.58±0.03 Å (Pd−Zn) and 2.76±0.03 Å (Pd−Pd) for the activated STD catalyst, indicating the formation of the PdZn phase during H_2_ activation (Supporting Information, Figure S6, Table S3).^8^

**Figure 3:**
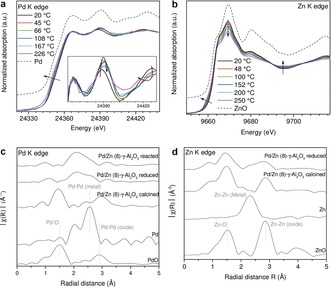
a),b) XANES spectra measured during in situ TPR experiments at a) Pd K and b) Zn K edges (RT to 250 °C; 5 % H_2_/He; heating rate 1 °C min^−1^; Pd/Zn (8)‐γ‐Al_2_O_3_). c),d) FT *k* ^2^‐weighted EXAFS spectra (not corrected for the phase shift) measured during in situ XAS experiments at c) Pd K and d) Zn K edges after calcination, reduction at 250 °C.

The STD process was then studied at high pressure using operando XAS and reaction products monitored by on‐line mass spectrometry (Supporting Information, Figure S4f). Spectra at Pd K edge were measured after the formation of methanol and DME had stabilized. The coordination numbers (CN, Supporting Information, Table S3, Figure S6) decreased from 7.1 (Pd‐Zn) and 3.6 (Pd‐Pd) in the H_2_‐activated catalyst to 5.5 (Pd‐Zn) and 2.6 (Pd‐Pd) during DME synthesis. The Pd‐Zn to Pd‐Pd CN ratio remained 2:1, in good agreement with the intermetallic bulk structure (Figure 1). Hence, the intermetallic PdZn phase remained stable during the STD process. A similar decrease in CN was reported by Tao et al.^16^ for a pure Pd catalyst under CO and attributed to the formation of small clusters on the catalyst surface. The CN decrease observed herein suggests similarly an increase in dispersion or an increase in surface area. DFT calculations were performed on (adsorbate‐induced) Pd segregation to investigate the surface structure under reaction conditions. They strongly indicate that the stability of the PdZn intermetallic compound prevents segregation of Pd, even in the presence of CO and OH (Supporting Information, Table S4; Figure S7).

DFT calculations furthermore confirmed prominent reaction paths for a high activity of PdZn alloys in CO hydrogenation to methanol, similar as for Cu. For this purpose, a PdZn L1_0_ slab model with (111) termination was used to calculate adsorption energies and transition states of the CO hydrogenation reaction (Figure 4 a,b). Calculations of PdZn(111) (red line) are compared to earlier calculations of CO hydrogenation on Cu(111) (black line) and Cu(211) (blue line) surfaces.^17^ Although these calculations were performed with a different functional (RPBE)^18^ than that used here (BEEF‐vdW),^19^ we note that calculations comparing RPBE and BEEF‐vdW for CO hydrogenation on Cu(211) revealed almost identical results.^20^ As shown in Figure 4 a, the strength with which intermediates and transition states are bound to the PdZn surface are strikingly similar to those calculated for Cu(211), which is believed to be the active site in CO hydrogenation.^17^, ^21^ In an attempt to understand why PdZn surfaces have common properties to that observed for Cu, we investigated the d‐projected density of states (DOS) and the corresponding d‐band center often used as a descriptor of surface reactivity.^22^ The d‐projected DOS of PdZn is compared with the DOS of Cu(111) and Pd(111) in Figure 4 c. The d‐states of Pd(111) are not fully filled, resulting in a d‐band center of −1.73 eV, similar to earlier reports.^23^ For PdZn, however, the d‐band is now completely filled and shifted downwards resulting in a d‐band center of −2.32 eV. This is again strikingly similar to the −2.40 eV obtained for Cu(111) (note that the d‐band of Cu(211) is even closer; −2.35 eV; Supporting Information, Figure S8). The local density of states of Pd in a PdZn alloy are thus similar to those of Cu, as also reported earlier.^24^

**Figure 4:**
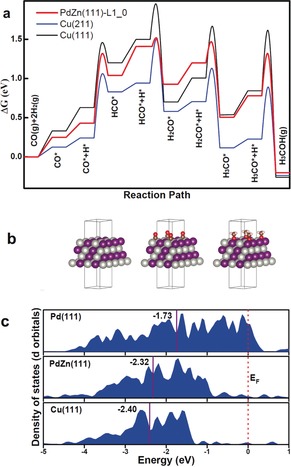
a) Gibbs‐free energy diagram for CO hydrogenation on Cu(111), Cu(211), and PdZn(111)‐L1_0_. Gibbs free energies are calculated for *T*=500 K, *p*(H_2_)=40 bar, *p*(CO)=10 bar, and *p*(CH_3_OH)=1 bar. Values for the Cu(111) and Cu(211) are taken from Ref. 16. b) PdZn(111)‐L1_0_ surface model (Pd light gray, Zn purple, O red, C brown), c) DOS calculated for Cu(111), Pd(111), and PdZn(111)‐L1_0_. Calculated d band centers are marked with dark red line.

In conclusion, uniform Pd/Zn‐based NPs with a reactive Pd^0^–Zn^2+^ interface ensured the formation of intermetallic PdZn NPs during in situ activation and provided the key building blocks for the methanol active component in bifunctional STD catalysts. High‐pressure operando XAS studies suggest intermetallic PdZn NPs as the methanol active species and DFT calculations confirm that these alloys are indeed highly active in CO hydrogenation to methanol. Enhanced stability and longevity can be achieved even at higher reaction temperature (300 °C) with STD catalysts based on such intermetallic PdZn particles, while ensuring both high product and low methane selectivities.

## Conflict of interest

The authors declare no conflict of interest.

## Supporting information

As a service to our authors and readers, this journal provides supporting information supplied by the authors. Such materials are peer reviewed and may be re‐organized for online delivery, but are not copy‐edited or typeset. Technical support issues arising from supporting information (other than missing files) should be addressed to the authors.

Supplementary
