# Supplementary material for: Supported Intermetallic PdZn Nanoparticles as Bifunctional Catalysts for the Direct Synthesis of Dimethyl Ether from CO‐Rich Synthesis Gas
Source: Angew Chem Int Ed Engl. 2019 Sep 19;58(44):15655–9. doi: 10.1002/anie.201906256 (PMC6856832; doi:10.1002/anie.201906256)
Supplement: Supplementary file 1 — Supplementary [file ANIE-58-15655-s001.pdf]

Angewandte Chemie
Eine Zeitschrift der Gesellschaft Deutscher Chemiker

Supporting Information

Supported Intermetallic PdZn Nanoparticles as Bifunctional Catalysts for the Direct Synthesis of Dimethyl Ether from CO-Rich Synthesis Gas

*Manuel Gentzen, Dmitry E. Doronkin, Thomas L. Sheppard, Anna Zimina, Haisheng Li, Jelena Jelic, Felix Studt, Jan-Dierk Grunwaldt, Jörg Sauer, and Silke Behrens**

anie_201906256_sm_miscellaneous_information.pdf

Table of Contents

Experimental procedures 3

Materials 3
Preparation of nanoparticle precursors 3
Preparation of bifunctional STD catalysts 3
Characterization 3
Catalytic testing 4
In situ and *operando* XAS experiments 4
DFT calculations 5

Results and Discussions 6

Elemental compositions and specific surface areas (**Table S1**) 6
CO conversions and C-based product selectivities in the direct synthesis of DME (**Table S2**) 6
Results of EXAFS analysis (**Table S3**) 6
Segregation energies for Figure S8 compared to the ordered intermetallic $L1_0$ structure (**Table S4**) 7
TEM images of calcined and spent Pd/Zn-based STD catalysts (**Figure S1**) 8
Carbon-based product selectivities in the STD process for Pd/Zn (8)-γ-Al_2O_3 and Pd/Zn (14)-γ-Al_2O_3 (**Figure S2**) 8
CO conversion of Cu/ZnO/Al_2O_3–based reference catalyst (250°C) over TOS (**Figure S3**) 9
Operando XANES spectra at Pd K and Zn K edges and on-line catalytic data of the Pd/Zn (8)-γ-Al_2O_3 catalyst (**Figure S4**) 10
Linear combination (LCA) fits of XANES spectra measured during reductive activation (**Figure S5**) 11
Fits of Pd K edge EXAFS spectra of the Pd/Zn (8)-γ-Al_2O_3 catalyst after reductive activation and STD reaction (**Figure S6**) 11
CO binding on PdZn system (**Figure S7**) 12
Calculated density of states and d band centers for Cu, Pd and PdZn (**Figure S8**) 12

Experimental Procedures

Materials: Unless specified otherwise, all steps of particle synthesis were carried out under argon atmosphere in anhydrous solvents using standard Schlenk techniques. All chemicals were used as received without any further purification. Diethylzinc ($(C_2H_5)_2Zn$, ≥ 52 wt.-% Zn) and anhydrous toluene (99.8 %) were purchased from Sigma Aldrich; palladium(II) acetylacetonate ($Pd(acac)_2$, ≥ 99.9 % trace metal basis) and γ-Al_2O_3 were obtained from Alfa Aesar. Gases (CO 3.7, H_2 6.0, Ar 6.0, N_2 6.0) were received from Air Liquide.

Preparation of nanoparticle precursors: Pd/Zn-based nanoparticles were synthesized *via* reductive stabilization. $(C_2H_5)_2Zn$ (5 mL of a 1.2 M solution in toluene) was added drop-wise over a period of 90 min to a stirred suspension of $Pd(acac)_2$ (4.9 g, 16 mmol) in toluene (325 mL) at 40 °C. The reaction was allowed to proceed at 40 °C for 24 h whilst stirring. Eventually, all volatile components were removed and the product was dried in vacuum. Pd/Zn-based nanoparticles (NPs) were received as a black powder.

Preparation of bifunctional STD catalysts: The Pd/Zn-based nanoparticles were suspended with γ-Al_2O_3 in toluene (50 mL) and stirred for 0.25 h at room temperature. The solvent was subsequently removed in vacuum, and the resulting bifunctional STD catalysts were calcined at 350 °C for 4 h (heating rate 110 °C/h) to remove any organic residues from nanoparticle synthesis. Finally, the catalysts were pelletized, crushed, and sieved to the fraction of 80 – 160 µm.

Characterization: For analysis of the NPs, a transmission electron microscope (TEM, FEI Tecnai F20 ST TEM, operating voltage 200 kV, equipped with a field emission gun and an EDAX EDS X-ray spectrometer (Si(Li) detecting unit, super ultra-thin window, active area 30 mm^2, resolution 135 eV (at 5.9 keV)) was used. Additionally, an aberration (image) corrected FEI Titan 80-300 (operated at 300 kV) equipped with an EDAX s-UTW EDX detector was employed for high resolution transmission electron microscopy (HRTEM) images of the used catalyst. For sample preparation, a droplet of the dispersed sample was deposited on amorphous carbon-coated Cu grids (400 mesh) and typically dried in air. For HRTEM of the spent catalysts, the samples were handled in an anoxic glove box and transferred to the TEM using a Gatan 648 vacuum transfer holder to avoid oxidation of the catalyst particles. The mean particle diameter was calculated based on TEM images by measuring the size of at least 100 particles. The analysis of the catalyst composition was carried out with a DSM 982 Gemini SEM (Zeiss corp., Germany, equipped with a Schottky-type thermal field emission cathode) coupled with EDX.

Powder x-ray diffraction (XRD) was carried out with a PANalytical X'Pert Pro X-ray diffractometer (Bragg-Brentano geometry with Cu Kα radiation and a Ni filter). The range between 5° and 120° was measured within 1 h. The diffraction patterns were compared to reference compounds from the Joint Committee of Powder Diffraction Standards (JCPDS) database. Reflections of the calcined and spent catalysts were assigned to the following JCPDS references: PdO (JCPDS 00-043-1024), Pd (JCPDS 01-087-0638), ZnO (JCPDS 01-089-0510) and γ-Al_2O_3 (JCPDS 00-010-0425) and the $L1_0$ PdZn phase (JCPDS 00-006-0620) (Fig. 1b). Crystallite sizes of the PdZn phase (L_{hkl}) were calculated for the reflection at 41.2° (2θ) (i.e. (111)) using the Scherrer equation

$$L_{hkl} = \frac{0.9\,\lambda}{\beta \cos\theta} \quad \text{(S1)}$$

where L_{hkl} is the particle size perpendicular to the (hkl) crystal plane, β the full width at half maximum, θ the Bragg angle, and λ (1.541 Å) the wavelength of the incident X-rays. LaB_6 (NIST) was used as a standard to account for the contribution of the instrumental broadening. To record XRD patterns of the used catalysts after the STD reaction, the samples were removed from the reactor in the anoxic glove box and deposited on the XRD sample holder which was eventually covered by Kapton foil. The broad peak at low Bragg angles (approx. 12-30° range) in the diffractograms of the spent catalysts (i.e., Pd/Zn (8)-γ-Al_2O_3 and Pd/Zn (14)-γ-Al_2O_3) results from the Kapton foil covering the catalyst samples during inert XRD measurements (Kapton sample cell window) and thus, is absent for the calcined catalyst samples.

Specific total surface areas of the bifunctional catalysts were determined using N_2-physisorption experiments in a Quantachrome Nova 2000e device. Prior to N_2-physisorption experiments, the calcined catalysts were degassed in vacuum at 230 °C for at least 12 h.

Temperature-programmed ammonia desorption (NH_3-TPD) was conducted in an Autochem 2950 device (Micromeritics). Prior to these experiments, the bifunctional catalysts were reduced in 2 vol.-% H_2 in Ar at 250 °C (heating rate 2 °C/min). Ammonia (1.221 ± 0.024 vol.-% NH_3 in He, 30 mL/min) was subsequently loaded after cooling to 100 °C. Physisorbed ammonia was initially removed in a He flow (30 mL/min) at 120 °C, and then ammonia desorption was confirmed from 120 to 750 °C (heating rate 4 °C/min) in He (30 mL/min) using a Cirrus 2 mass spectrometer (MKS).

Catalytic testing: The performance of the bifunctional catalysts was evaluated using a continuously operated laboratory scale plant with a plug flow reactor. Inlet gases (obtained from Air Liquide: CO (N37), H_2(6.0), Ar(6.0), N_2(6.0)) were dosed using individual mass flow controllers (MFC, Bronkhorst). The reaction mixture was diluted with inert gas (70 vol.-% Ar/N_2) to avoid the formation of hotspots in the reactor. Analysis of outlet gases was carried out with a gas chromatograph (GC, Hewlett Packard 6890) equipped with two columns (RESTEK RT®-U-Bond, RESTEK RT-M sieve) and two detectors (thermal conductivity detector, flame ionization detector). 2 g calcined catalyst were placed in the fixed bed reactor between two layers of silicon carbide followed by *in situ* activation (activation procedure: 1) heating (17 °C/h) in 2 vol.-% H_2 in Ar (100 mL_{NTP}/min) to 200 °C which was maintained for 1 h, 2) heating (17 °C/h) to 240 °C which was maintained for 1 h, and 3) heating (10 °C/h) in pure H_2 (50 mL_{NTP}/min) to 250 °C which was maintained for 1 h). After applying the reaction pressure (50 bar), the reactant gases were fed into the reactor with a total flow of 50 mL_{NTP}/min and a composition of Ar : N_2 : H_2 : CO = 5 : 2 : 1.5 : 1.5. The temperature was increased every 6 h by 10 °C within 1h until the final temperature of 300 °C was reached. A maximum deviation of the reaction temperature by ± 0.2 K over the entire length of the catalyst bed was ensured by monitoring the temperature profile with a thermocouple in a guiding sleeve in the middle of the reactor. Stability with time on stream (TOS) was tested at 250°C over a period of 270 h and 260 h for Pd/Zn (8)-γ-Al_2O_3 and for the Cu/ZnO/γ-Al_2O_3–based reference catalyst, respectively. CO conversion and product selectivities were evaluated according to the following equations:

$$X_{CO} = \frac{\dot{N}_{CO,0} - \dot{N}_{CO}}{\dot{N}_{CO,0}} \cdot 100 \qquad \text{(S2)}$$

where $\dot{N}_{CO,0}$ is the molar CO flow at the reactor inlet, $\dot{N}_{CO}$ the molar CO flow at reactor outlet and X_{CO} the CO conversion (%).

$$S_i = \frac{\xi_i(\dot{N}_i - \dot{N}_{i,0})}{\dot{N}_{CO,0} - \dot{N}_{CO}} \cdot 100 \qquad \text{(S3)}$$

where S_i is the selectivity of component *i*, and $\dot{N}_i$ and $\dot{N}_{i,0}$ molar flow of component *i* at reactor outlet and at reactor inlet, respectively, and ξ_i is the number of carbon atoms in a molecule of component i.

***In situ and operando XAS experiments*:** *In situ* X-ray absorption spectra (XAS) in terms of the X-ray absorption near-edge structure (XANES) and extended X-ray absorption fine structure (EXAFS) were recorded at the CAT-ACT beamline at the Synchrotron Radiation Source at KIT (Karlsruhe) in fluorescence mode using a PIPS diode.[1] The X-ray energy was varied using a double crystal monochromator with a pair of Si (111) and Si (311) crystals to scan energies around Zn K and Pd K edges, respectively. Higher harmonics were rejected by a pair of Si or Pt/Ir coated mirrors for Zn K or Pd K edges. The incident beam was focused with the second mirror but no slits were available to shape the beam in front of the first ionization chamber. Due to this and the long optical path between the optics hutch and the experimental hutch, the resulting beam size on the sample at 24350 eV could not be precisely controlled and could exceed 1 mm x 1 mm (vertical x horizontal) and at Pd K edge approach 1.5 mm x 3 mm. The catalyst (approx. 6 mg, 80-160 µm sieve fraction) was placed in an *in situ* microreactor (quartz capillary with a plug flow reactor geometry, 1.5 mm diameter, 20 µm wall thickness, catalyst bed length approx. 5 mm) between two quartz wool plugs and mounted horizontally over a hot air blower (FMB Oxford GSB-1300).[2] The temperature was calibrated using readings of an external thermocouple at the top and the bottom of the capillary microreactor in a separate test without X-ray beam. He or 5 vol.% H_2/He were dosed using individual mass flow controllers at a flow rate of 50 mL_{NTP}/min, and the capillary microreactor was pressurized up to 20 bar by means of a Back Pressure Regulator (BPR, Bronkhorst). First, XANES and EXAFS spectra were recorded at 25 °C in He flow, afterwards the gas mixture was switched to 5 % H_2/He (50 mL_{NTP}/min, ambient pressure) and a temperature programmed reduction (TPR) was performed. For this purpose, XANES spectra were recorded continuously during heating to 250 °C at a ramp rate of 1 °C/min. After reaching 250 °C, the catalyst was kept for 60 min in 5% H_2/He, after which another set of XANES and EXAFS spectra were recorded (5 % H_2/He, 250 °C). The reduction experiments were repeated twice while recording either spectra at Pd K edge (the sieved catalyst was used as received) or at Zn K edge (the catalyst was diluted by mechanically mixing with γ-Al_2O_3 (catalyst/γ-Al_2O_3 ratio 1:1) prior to pressing and sieving to diminish the incident beam self-absorption effect). After the TPR experiment at the Pd K edge, the reaction conditions of the direct DME synthesis were applied to the *in situ* microreactor (15 vol.-% H_2, 15 vol.-% CO in He, 250 °C, 18 mL_{NTP}/min, 20 bar) and maintained for one hour. The product formation was qualitatively monitored thanks to the elevated pressure by a Pfeiffer Vacuum OmniStar GSD320 mass spectrometer (m/z 45 and 31 were observed for DME and methanol, respectively). The possibility to actually observe the reaction products and to prove that the catalyst actually produced DME at the same time makes the study the first *operando* (*i.e.*, performed on a working material)[3] study of DME catalysts. During *in situ* low pressure (1 bar) measurements hardly any methanol or DME could be detected (Fig. S6c); the signals of the products appeared only after pressurizing to 5 bar with methanol prevailing over DME (we neglect difference in ionization probabilities due to similar chemical nature of methanol and DME). Only after applying 20 bar, DME became the main reaction product as demonstrated by the MS data. Thus, performing measurements at high pressure is a prerequisite for an *operando* study of methanol or DME synthesis catalysts.

During the test under reaction conditions, XANES spectra were continuously recorded, an additional EXAFS spectrum at the Pd K edge was recorded after 1 h TOS when concentrations of DME and methanol were stabilized. The spectra were normalized, and background subtracted to extract EXAFS using the ATHENA program from the IFFEFIT software package.[4] The spectra of the catalyst measured at Zn K edge were corrected for incident beam self-absorption using the Fluo algorithm built in the ATHENA program.[4] The k^1-, k^2-, and k^3-weighted EXAFS functions were Fourier transformed in the k range of 3.0 – 11 $Å^{-1}$ and multiplied by a Hanning window with sill size of 1 $Å^{-1}$ (not corrected for phase shift). The structural models were based on bulk Pd metal (Inorganic Crystal Structure Database, ICSD collection code 52251) and PdZn alloy (ICSD collection code 105752). The coordination numbers and interatomic distances were received by fitting one coordination shell for the Pd-Zn neighbors and a second coordination shell for the Pd-Pd neighbors. The structure refinement was performed using ARTEMIS software (IFFEFIT).[4] For this purpose, the corresponding theoretical backscattering amplitudes and phases were calculated by FEFF 6.0.[5] The theoretical data were then adjusted to the experimental spectra by a least square method in R space between 1.5 and 2.9 $Å^{-1}$. First, the amplitude reduction factors (S_0^2 = 0.58) were calculated using the Pd foil reference spectrum, and then the coordination numbers (CN), interatomic distances (d), energy shift (δE_0) and mean square deviation of interatomic distances (σ^2) were refined. The absolute misfit between theory and experiment was expressed by ρ. Fitting was done in iterative way several times constraining CNs of Zn or Pd until reaching the lowest ρ. The number of fitting parameters was always lower than the number of independent points.

The fractions of ZnO and Zn^0 species in the *in situ* XANES spectra were determined by a Linear Combination Analysis (LCA) using spectra of the calcined catalyst corrected for the incident beam self-absorption (an *ex situ* spectrum of the calcined sample recorded in transmission mode confirmed ZnO structure) and Zn foil reference compounds in the fitting range 9652 – 9702 eV. No additional constraints were used / required to achieve the linear combination fit. R-factor obtained during the fit was 0.0004 (0.04%), and the error bars were ±0.6%. On one hand, the obtained fit is rather good implying that Zn foil spectrum can be used to represent the spectrum of reduced Zn species, however, on the other hand, it has been shown that XANES spectra of bulk metals do not necessarily represent well spectra of the nanoparticles.[6] This is especially true if intermetallic compound is formed. Hence, we cannot be completely sure that Zn foil spectrum represents the reduced Zn species well and, therefore, in the text we use very conservative ±10% error bars for the LCA.

In addition, LCA was also performed on the Pd K edge XANES spectra to evaluate relative spectral changes during the reductive activation of the catalyst. The spectral changes were very small (Fig. 3a). As no spectra of reference compounds could be used, the first spectrum before the heating (in H_2/He at 20 °C) and the spectrum of the activated catalyst were used as internal references instead.

DFT calculations: DFT calculations have been performed using the Vienna Ab Initio Simulation Package (VASP)[7] in connection with the Atomic Simulation Environment (ASE).[8] The Bayesian Error Estimation Functional with van der Waals correlations (BEEF-vdW),[9] that is performing well for adsorption energies and transition states on transition metal surfaces[10] has been used. The plane-wave cutoff was 500 eV. The (111) facet of PdZn surfaces in the $L1_0$ structure was modeled by periodic slabs consisting of 2 x 2 atoms in the x and y direction and four layers in the z-direction with 16 Å of vacuum between the slabs. Only the top two layers were allowed to relax while the other two layers where kept at their bulk position. The Brillouin zone was sampled using a 6 x 6 x 1 Monkhorst-Pack k-point grid.[11] Transition state searches were performed using the nudged elastic band (NEB) method.[12]

Results and Discussion

Table S1. Elemental composition, specific total surface area A_{BET} and of the bifunctional Pd/Zn-γ-Al_2O_3 catalysts

Catalyst	Pd (wt.-%)	Zn (wt.-%)	γ-Al_2O_3 (wt.-%)	A_{BET} (m²/g)	Desorbed NH_3[a] (µmol$_{NH3}$/g$_{cat}$)
**Pd/Zn (8)-γ-Al_2O_3**	8.4	19.8	50.2	154	135
**Pd/Zn (14)-γ-Al_2O_3**	14.3	26.2	31.9	126	132

[a] NH_3 with a desorption temperature below 500 °C, which is attributed to weak and medium acidic sites of γ-Al_2O_3.

The total specific surface areas of 154 m^2/g (Pd/Zn (8)-γ-Al_2O_3) and 126 m^2/g (Pd/Zn (14)-γ-Al_2O_3) are lower than the one of the pristine γ-Al_2O_3 support (221 m²/g). It should be noted that the γ-Al_2O_3 support represents only 50.2 wt.-% (*i.e.*, Pd/Zn (8)-γ-Al_2O_3) and 31.9 wt.-% (*i.e.*, Pd/Zn (14)-γ-Al_2O_3) of the bifunctional catalysts, which amounts to 111 m²/g and 70.5 m²/g, respectively. Hence, the difference in specific surface area is ascribed to the contribution of the supported Pd/Zn-based NPs. For γ-Al_2O_3, the weak to medium acidic sites are expected to exhibit a superior dehydration activity compared to strong acidic sites.[13]

Table S2. CO conversion and carbon-based product selectivities in the direct synthesis of DME for the Pd/Zn catalysts (Reaction conditions: temperature range 250 °C to 300 °C, 50 bar, feed composition H_2 : CO : Ar : N_2 = 3 : 3 : 10 : 4, total flow 50 ml$_{NTP}$/min)
X_{Eq} Equilibrium CO conversion under the chosen reaction conditions according to the reation equation: 3 CO + 3 $H_2 \rightleftharpoons CH_3OCH_3 + CO_2$

Pd/Zn (8)-γ-Al_2O_3

Reaction temperature (°C)	X_{CO}(%)	X_{Eq}(%)	S_{DME}(%)	S_{CO2}(%)	S_{MeOH}(%)	S_{CH4}(%)
250	13.6	66.4	64.8	31.5	3.0	0.7
260	21.6	60.5	64.9	31.8	2.7	0.6
270	30.5	54.1	64.7	32.4	2.2	0.7
280	35.5	47.3	63.6	33.6	2.0	0.8
290	36.8	40.4	63.7	33.0	1.9	1.4
300	33.5	33.6	61.6	33.6	2.0	2.4

Pd/Zn (14)-γ-Al_2O_3

Reaction temperature (°C)	X_{CO}(%)	X_{Eq}(%)	S_{DME}(%)	S_{CO2}(%)	S_{MeOH}(%)	S_{CH4}(%)
250	19.4	66.4	63.9	27.1	8.9	0.1
260	24.2	60.5	65.3	28.5	5.9	0.2
270	30.5	54.1	66.1	29.8	3.6	0.3
280	34.0	47.3	66.2	30.4	2.7	0.5
290	35.4	40.4	65.1	31.3	2.3	0.8
300	33.8	33.6	63.8	31.8	1.2	1.5

Table S3. Results of the EXAFS analysis of the spectra of the reduced and reacted Pd/Zn (8)-γ-Al_2O_3 (fit in *R* space between 1.5 and 3.0 Å^{-1}, S_0^2 = 0.58).

Sample	*d* Pd-Zn (Å)	CN (Zn)	σ^2 (Zn) (10^{-3} Å^2)	*d* Pd-Pd (Å)	CN (Pd)	σ^2 (Pd) (10^{-3} Å^2)	δE_0 (eV)	ρ (%)
Pd foil	-	-	-	2.73±0.003	12	4.3±0.5	3.1±0.5	0.2
PdZn (theoretical)	2.65	8	-	2.90	4	-	-	-
Reduced	2.58±0.03	7.1	13.9±2.3	2.76±0.03	3.6	9.6±3.2	0.4±4.2	0.3
Reacted	2.56±0.05	5.5	7.5±1.8	2.79±0.09	2.6	10.6±9.8	-3.8±8.8	1.1

Table S4. Segregation energies for the systems given in Figure S6 compared to the ordered intermetallic $L1_0$ structure (Figure S6a). CO and OH induced segregation energies are also given along with CO and OH binding energies relative to CO and H_2O-1/2H_2 gas phase, respectively.

(eV)	$L1_0$	Surface Pd-Zn rearrangement	Pd part. segregation	Pd full segregation	Zn part. segregation	Zn full segregation
Segregation energy *E*-*E*($L1_0$)	0.00	0.40	0.88	2.53	0.55	1.60
CO induced segregation	0	0.22	0.48	2.42	0.51	2.24
OH induced segregation	0	0.36	1.26	3.35	0.50	1.37

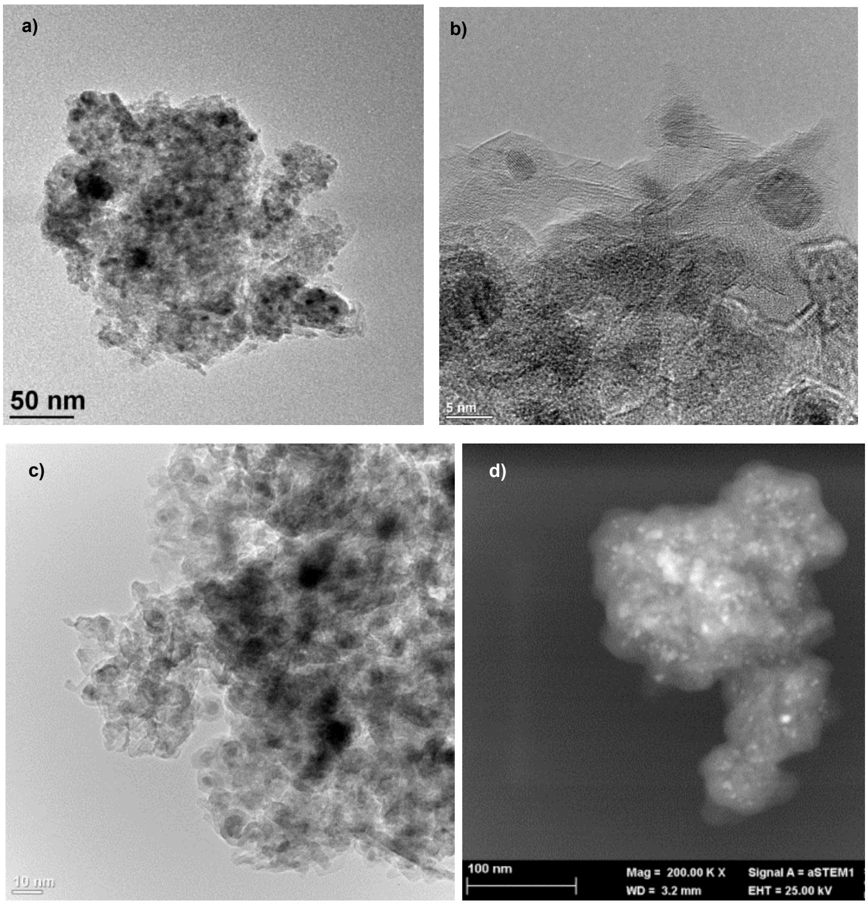

Figure S1. TEM images of the a) calcined and b,c) spent Pd/Zn NP-derived STD catalyst. d) SEM image (STEM/HAADF detector) of the spent STD catalyst (Pd/Zn (8)-γ-Al_2O_3).

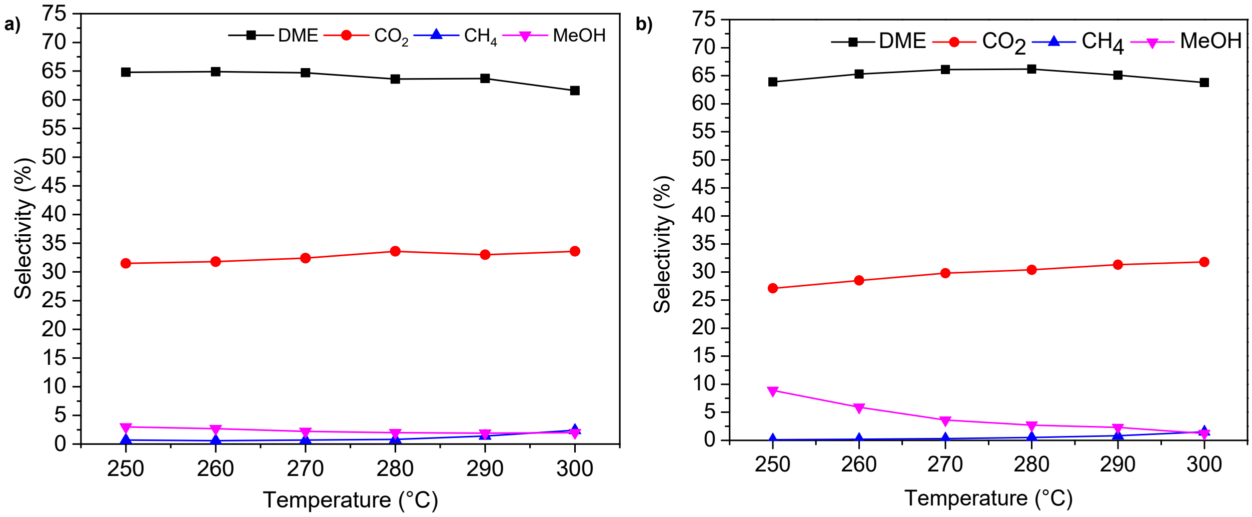

Figure S2. Carbon-based product selectivities in the direct synthesis of DME from CO-rich synthesis gas for temperatures between 250 °C and 300 °C (50 bar, H_2:CO = 1, 70 vol.-% inert gases) for a) Pd/Zn (8)-γ-Al_2O_3 and b) Pd/Zn (14)-γ-Al_2O_3.

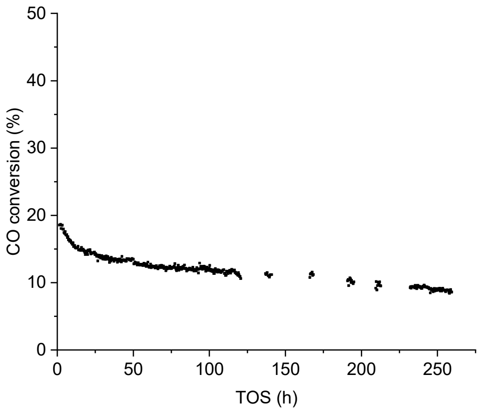

Figure S3. Decrease in CO conversion for a nanoparticle-derived, Cu/ZnO/Al_2O_3–based reference catalyst at 250°C over TOS.

In general, catalyst deactivation of conventional Cu/ZnO-based methanol catalysts has been ascribed to multiple mechanisms such as carbon deposition,[14] sintering,[15] and the formation of a dense ZnO overlayer on the Cu nanoparticles.[16] A Cu/Zn-based catalyst of the conventional methanol catalyst composition was prepared *via* a similar colloidal approach and used as a reference in the STD reaction. The catalytic tests revealed a decrease in CO conversion from originally 19% to 9% after testing for 260 h TOS. Copper has a low Hüttig temperature which is reflected by a relatively low melting point (i.e., bulk Cu 1083 °C), and therefore, Cu catalysts are operated typically at relatively low temperatures (240 to 260 °C). However, our Cu/ZnO-based reference catalyst revealed a constant deactivation even at 250°C with a decrease of 53% in CO conversion during the first 260 h TOS (Figure S3).

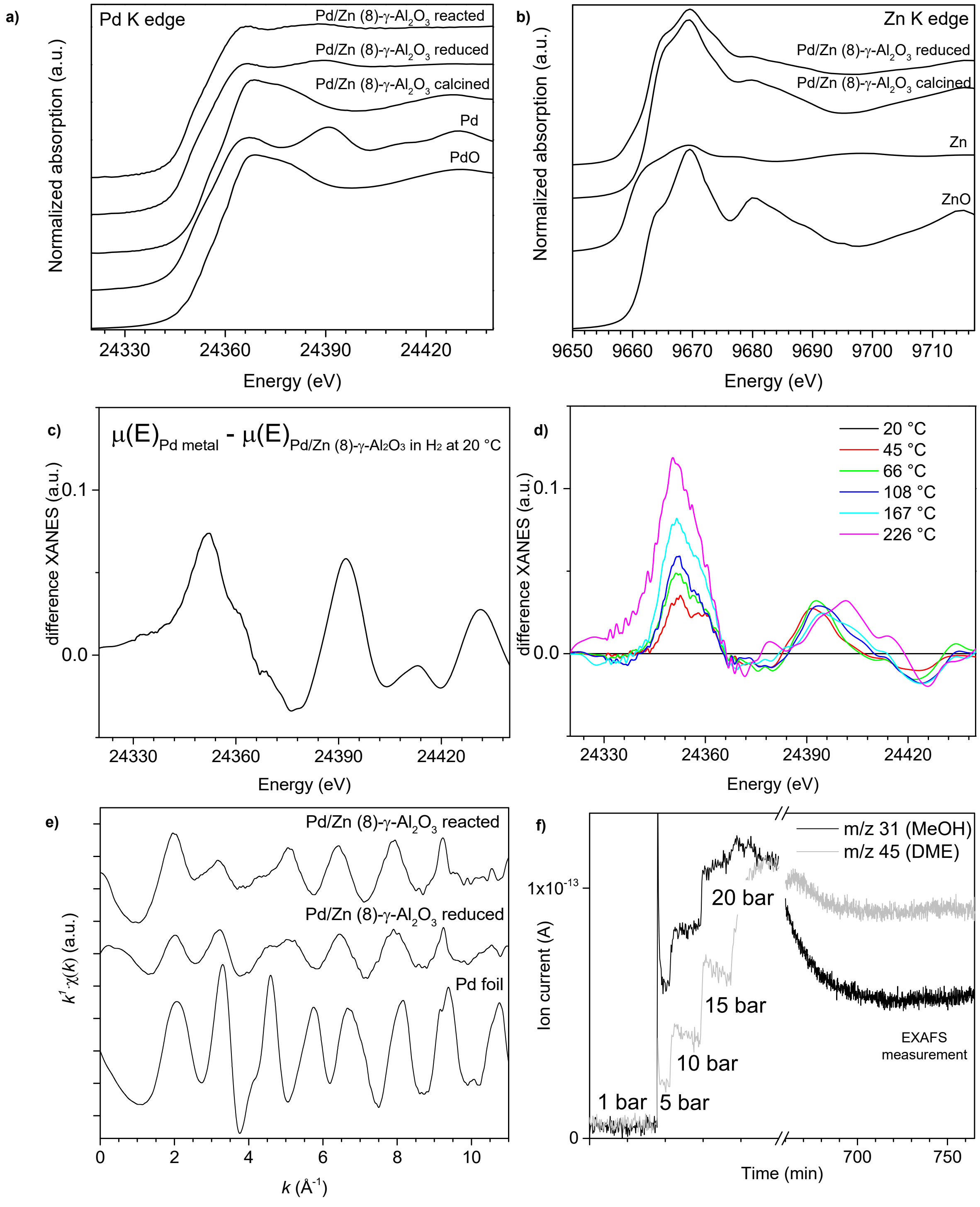

Figure S4. XANES spectra at a) Pd K and b) Zn K edges of the calcined Pd/Zn (8)-γ-Al_2O_3 catalyst, the reduced catalyst at 250 °C, and after 1 hour after applying reaction conditions (only at Pd K edge, 15 vol.-% H_2, 15 vol.-% CO in He, 250 °C, 20 bar) with the corresponding reference spectra. c) Difference Pd K edge XANES (ΔXANES) spectra catalyst obtained by subtracting the first spectrum measured at 20 °C (PdH_x phase) from the metallic Pd (Pd foil) spectrum. d) Difference Pd K edge XANES (ΔXANES) spectra measured *in situ* during TPR of the Pd/Zn (8)-γ-Al_2O_3 catalyst obtained by subtracting the first spectrum measured at 20 °C (PdHx phase). e) k^1-weighted $\chi(k)$ functions (extracted fine structure in *k*-space) of the reduced Pd/Zn (8)-γ-Al_2O_3 catalyst at 250 °C, and after 1 hour after applying reaction conditions. f) Ion currents of methanol and DME most abundant fragment ions recorded at the outlet of the *in situ* microreactor with bifunctional Pd/Zn (8)-γ-Al_2O_3 catalysts during *operando* EXAFS study (1-20 bar, H_2:CO = 1, 70 vol.-% He, 250 °C).

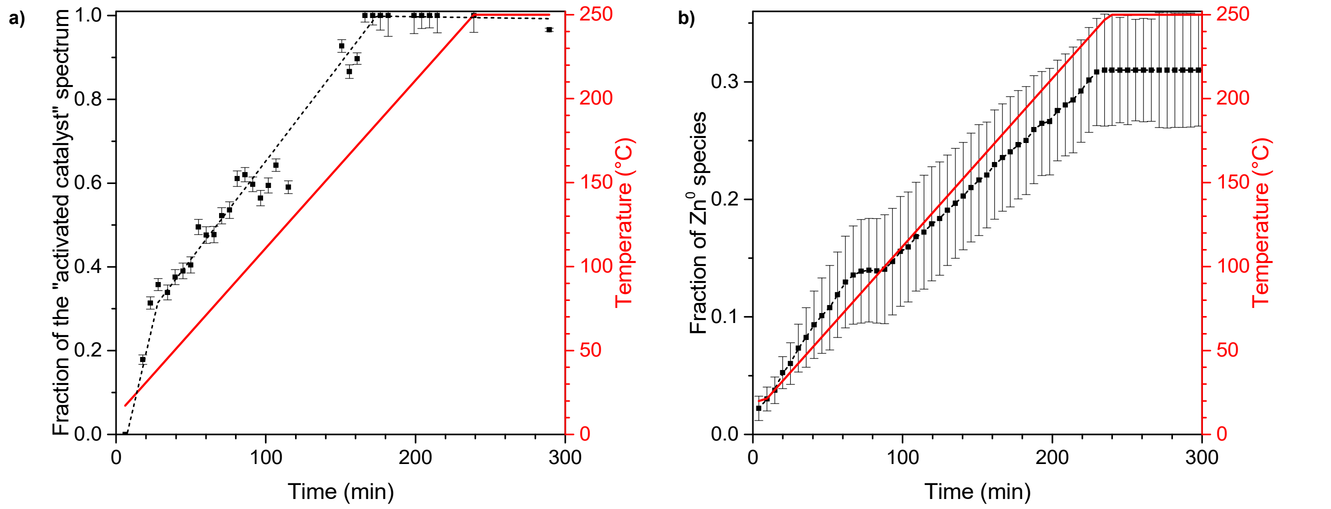

Figure S5. Linear combination (LCA) fits of XANES spectra measured during reductive activation of the Pd/Zn (8)-γ-Al_2O_3 catalyst at a) Pd K and b) Zn K edges. Internal references (the first and the last spectra measured during the activation) are used for the Pd K edge data, ZnO and Zn^0 (Zn foil) spectra are used for the Zn K edge data.

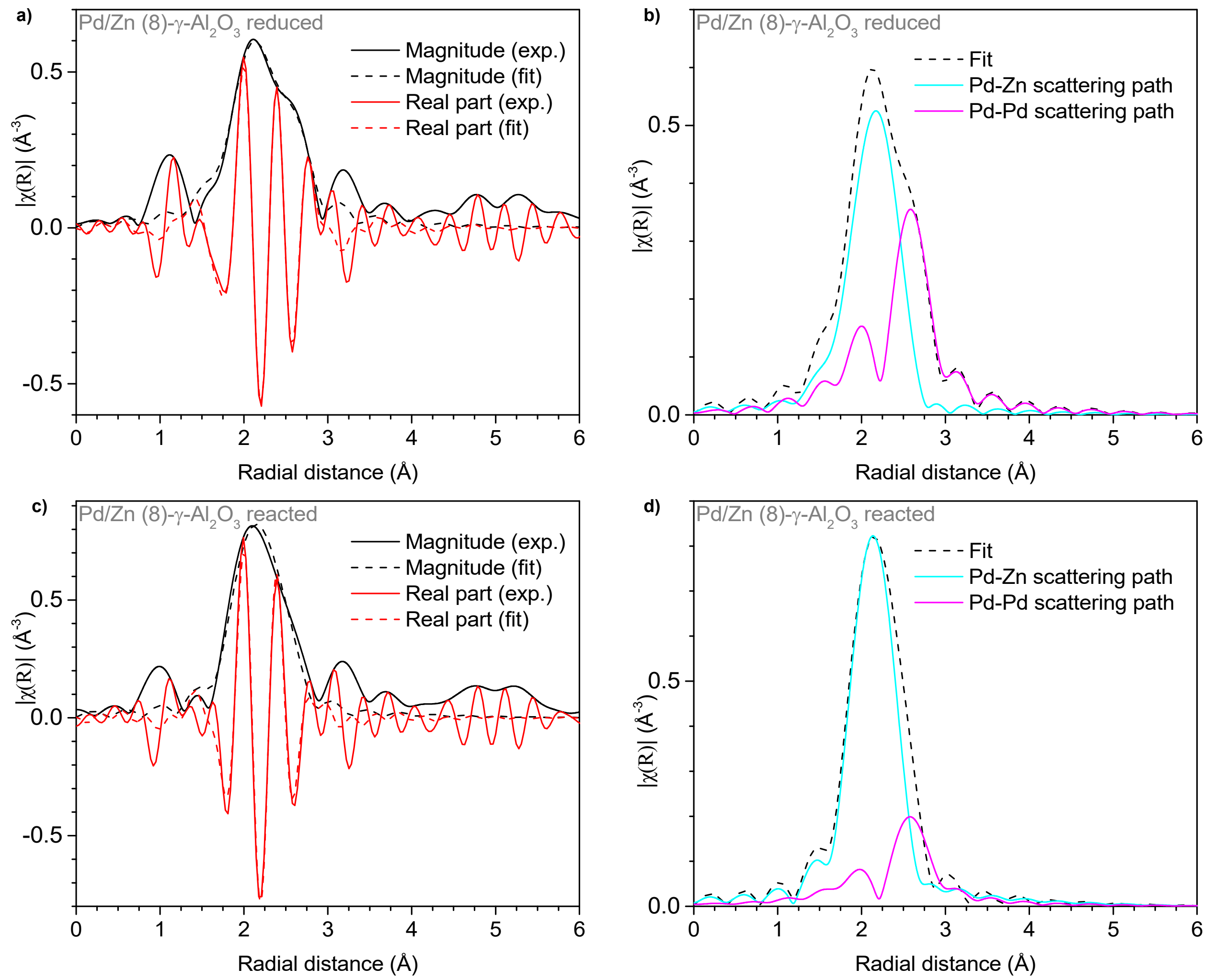

Figure S6. Fits of Pd K edge EXAFS spectra of the Pd/Zn (8)-γ-Al_2O_3 catalyst a), b) after reductive activation and c), d) after STD reaction. Parts b) and d) highlight contributions of individual scattering paths (Zn and Pd nearest neighbors) to the fits.

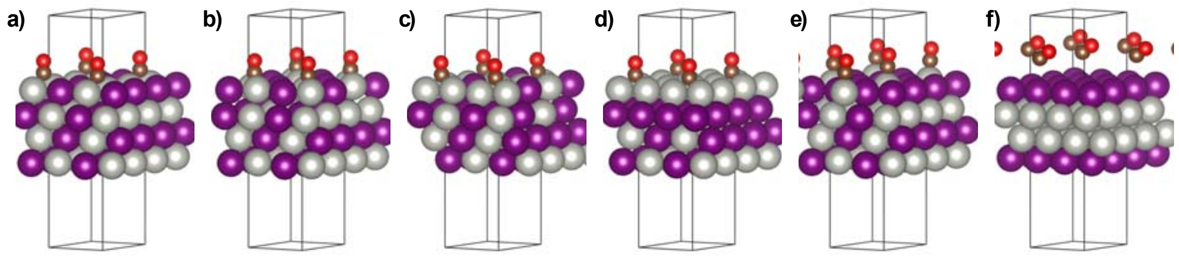

Figure S7. CO binding on PdZn systems: a) most stable $L1_0$ structure, b) surface Pd-Zn atom rearrangement, c) partial Pd segregation at the surface, d) full Pd segregation at the surface, e) partial Zn segregation at the surface, and f) full Zn segregation at the surface (Pd light gray, Zn purple, O red, C brown).

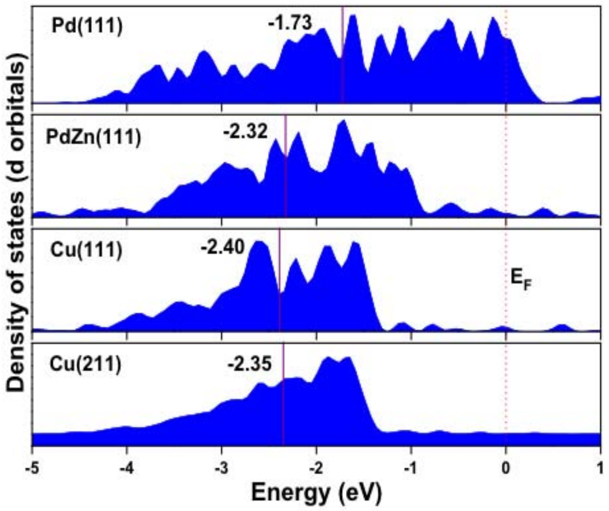

Figure S8. Density of states calculated for Cu(111), Cu(211), Pd(111) and PdZn(111)-$L1_0$. Calculated d band centers are marked with dark red line.

References

[1] A. Zimina, K. Dardenne, M. A. Denecke, D. E. Doronkin, E. Huttel, H. Lichtenberg, S. Mangold, T. Pruessmann, J. Rothe, T. Spangenberg, R. Steininger, T. Vitova, H. Geckeis, J.-D. Grunwaldt, *Review of Scientific Instruments* **2017**, *88*, 113113.
[2] a) J. D. Grunwaldt, M. Caravati, S. Hannemann, A. Baiker, *Physical Chemistry Chemical Physics* **2004**, *6*, 3037-3047; b) J.-D. Grunwaldt, N. v. Vegten, A. Baiker, *Chemical Communications* **2007**, 4635-4637.
[3] a) M. A. Bañares, M. O. Guerrero-Perez, J. L. G. Fierro, G. G. Cortez, *Journal of Materials Chemistry* **2002**, *12*, 3337-3342; b) B. M. Weckhuysen, *Physical Chemistry Chemical Physics* **2003**, *5*, 4351-4360.
[4] B. Ravel, M. Newville, *Journal of Synchrotron Radiation* **2005**, *12*, 537-541.
[5] J. J. Rehr, R. C. Albers, *Reviews of Modern Physics* **2000**, *72*, 621-654.
[6] D. Bazin, J. J. Rehr, *J. Phys. Chem. C* **2011**, *115*, 23233–23236.
[7] G. Kresse, J. Furthmüller, *Physical Review B* **1996**, *54*, 11169-11186.
[8] S. R. Bahn, K. W. Jacobsen, *Computing in Science & Engineering* **2002**, *4*, 56-66.
[9] J. Wellendorff, K.T. Lundgaard, A. Møgelhøj, V. Petzold, D.D. Landis, J.K. Nørskov, T. Bligaard, K.W. Jacobsen, *Phys. Rev. B* **2012**, 85, 235149
[10] a) J. Wellendorff, T. L. Silbaugh, D. Garcia-Pintos, J. K. Nørskov, T. Bligaard, F. Studt, C. T. Campbell, *Surface Science* **2015**, *640*, 36-44; b) S. Mallikarjun Sharada, T. Bligaard, A. C. Luntz, G.-J. Kroes, J. K. Nørskov, *The Journal of Physical Chemistry C* **2017**, *121*, 19807-19815.
[11] H. J. Monkhorst, J. D. Pack, *Physical Review B* **1976**, *13*, 5188-5192.
[12] G. Henkelman, B. P. Uberuaga, H. Jónsson, *The Journal of Chemical Physics* **2000**, *113*, 9901-9904.
[13] M. Mollavali, F. Yaripour, H. Atashi, S. Sahebdelfar, *Industrial & Engineering Chemistry Research* **2008**, *47*, 3265-3273.
[14] A. Aguayo, J. Ereña, I. Sierra, M. Olazar, J. Bilbao, *Catal. Today* **2005**, *106*, 265-270.
[15] H. Kung, Catal. Today 1992, 11 (4), 443-453.
[16] T. Lunkenbein, F. Girgsdies, T. Kandemir, N. Thomas, M. Behrens, R. Schlögl, E. Frei, *Angew. Chem. Int. Ed.* **2016**, 55 (41), 12708-12712.
